# A Real-Time Smart Sensor for High-Resolution Frequency Estimation in Power Systems

**DOI:** 10.3390/s90907412

**Published:** 2009-09-15

**Authors:** David Granados-Lieberman, Rene J. Romero-Troncoso, Eduardo Cabal-Yepez, Roque A. Osornio-Rios, Luis A. Franco-Gasca

**Affiliations:** 1 HSPdigital Research Group, División de Ingenierías, Campus Irapuato-Salamanca, Universidad de Guanajuato / Carr. Salamanca-Valle km 3.5+1.8, Comunidad de Palo Blanco, 36700 Salamanca, Guanajuato, Mexico; E-Mails: granlieber@hspdigital.org (D.G.-L.); troncoso@hspdigital.org (R.J.R.-T.); 2 Facultad de Ingeniería, Campus San Juan del Río, Universidad Autónoma de Querétaro / Río Moctezuma 249, Col. San Cayetano, 76807 San Juan del Río, Querétaro, Mexico; E-Mail: raosornio@hspdigital.org; 3 LabCASD, CIATEQ, Calz. del Retablo 150, Col. Fovissste, 76150 Querétaro, Qro., Mexico; E-Mail: luis.franco@ciateq.mx

**Keywords:** smart sensor, frequency estimation, chirp *z*-transform, high resolution, power systems

## Abstract

Power quality monitoring is a theme in vogue and accurate frequency measurement of the power line is a major issue. This problem is particularly relevant for power generating systems since the generated signal must comply with restrictive standards. The novelty of this work is the development of a smart sensor for real-time high-resolution frequency measurement in accordance with international standards for power quality monitoring. The proposed smart sensor utilizes commercially available current clamp, hall-effect sensor or resistor as primary sensor. The signal processing is carried out through the chirp *z*-transform. Simulations and experimental results show the efficiency of the proposed smart sensor.

## 1. Introduction

Power quality monitoring is a theme in vogue and accurate frequency measurement of the power line is a major issue. This problem is particularly relevant for power generating systems as shown in the work of Xue and Yang [1], since the generated signal must comply with restrictive international standards. The international standard CEI/IEC 61000-4-30 [2] specifies that the frequency measurement for class-*A* performance power systems must be obtained every 10 s. the measurement time intervals shall be non-overlapping, and the measurement uncertainty shall not exceed ±0.01 Hz. This standard demands performance features that are not easily met by most commercially available measurement equipment, requiring expensive instrumentation systems to comply with specifications [3]. Regardless of the cost, the measured frequency from these systems cannot be easily integrated into the power generation process to provide the feedback in order to accurately control the frequency at the output generated signal. Moreover, the output signal is generally embedded in noise, which increases the difficulty of the monitoring process within the required accuracy. Therefore, there is a clear need for a simple and inexpensive way to accurately measure the frequency of the line on power generating systems.

Accurate frequency detection of a periodic signal embedded in noise is a problem that has been largely studied for several applications. Many methodologies [4–7] have been proposed for tackling this problem, most of them relaying on Fast Fourier Transform (FFT) computation. Unfortunately, as it has been thoroughly shown, the FFT offers a fast processing engine, but its performance and resolution heavily depend on the signal-to-noise ratio (SNR) and the number of samples from the analyzed signal, jeopardizing its compliance on certain application requirements as those of frequency monitoring in power systems. Regarding this subject, Cheng and Fang [8] presented a methodology for frequency measurement in power systems combining the FFT with a quadratic interpolation technique in order to improve its precision and resolution, obtaining accuracies of 99.74% with estimation errors around 0.1 and 0.2 Hz. Qing-Qiang *et al.* [9] presented an algorithm for measuring frequency by sampling current signals utilizing rigorous mathematics, Newton iterative techniques, and Taylor series expansions, obtaining measurement errors around 1.0 Hz. López *et al.* [10] proposed a methodology for power-system frequency measurement based on the monitoring of the voltage signal, applying statistical computations, and a weighting factor to prefiltered samples in order to reduce the estimation error, reaching uncertainties of 0.025 Hz and 0.035 Hz. Xue and Yang [1] presented a supervised Gauss-Newton algorithm for power system frequency estimation applying a recursive discrete Fourier transform (DFT), a zero-crossing method, and an infinite-impulse response (IIR) filter, obtaining estimation errors around 0.1 Hz. Bellini *et al*. [11] use the parameters of the Zoom-FFT algorithm to increases the frequency resolution, keeping constant the computational cost, for detecting rotor faults on induction motors; the obtained resolution applying this technique is 0.1 Hz. The chirp *z*-transform (CZT) has a higher frequency resolution than the FFT for the same number of input samples regardless SNR as shown in the works of Feng-Xiang *et al.* [12], and Nguyen and Li [13]. In [12] an algorithm for accurate frequency estimation is proposed based on a recurrent CZT computation that narrows the frequency bandwidth at each iteration, until the desired resolution is reached. In [13] a *z*-transform signal model combined with nonlinear postfiltering is proposed for estimating the operating frequency in a power system utilizing simulation studies. Unfortunately, the complexities in these methodologies difficult the real time and online frequency estimation in power systems, compromising the compliance of international standards. From the exposed above, it is evident the necessity for a frequency estimation methodology that combines real-time processing with high-resolution results. A promising approach to overcome the needs on performance and cost is the smart sensor, which utilizes a standard sensor and includes in its functionalities signal processing, communication, and integration capabilities. The term “smart sensor” is employed according to the functionality classification given by Rivera *et al.* [14], from the definitions of the Institute of Electrical and Electronics Engineers [15,16].

The novelty of this work is the development of a smart sensor for real-time high-resolution frequency measurement, in accordance with the international standard CEI/IEC 61000-4-30 [2] for power quality monitoring. The proposed smart sensor can utilize a commercially available current clamp, a hall-effect sensor or a resistor as primary sensor of the signal in which the frequency measurement is to be performed. Besides, the CZT that computes the power spectrum of the analyzed signal for the high-resolution frequency estimation is implemented into a low-cost field-programmable gate array (FPGA) with a processing time of 1.0078 s and readouts every 2 s. The CZT implemented algorithm is performed in the real domain, contrary to [12], to achieve the desired processing time. Several cases of study, including simulations and experimental results, are presented to show the effectiveness and performance of the proposed smart sensor.

## 2. Theoretical Background

**Figure 1.**
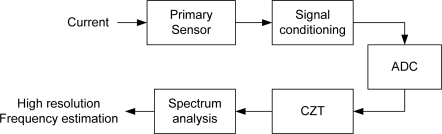
Block diagram of the frequency-monitoring smart sensor.

Figure 1 shows the block diagram of the proposed smart sensor for frequency monitoring. The system uses a standard primary sensor (current clamp, hall-effect sensor or resistor) to measure the signal under analysis. Signal conditioning is then applied. Afterwards, the sensed signal is converted to digital in the analog-to-digital converter (ADC). The quantized information is then processed utilizing the CZT for obtaining a high-resolution frequency spectrum. Finally, the resulting spectrum is analyzed in order to determine the component with the highest magnitude that provides the signal frequency with the required resolution for meeting the standard demands.

### 2.1. Chirp-Z Transform

The CZT *X(k)* of an *N*-point sequence *x(n)* for *n* = 0, 1, 2, …, *N−1* is given by Equation (1). In Equation (1), *X(k)* allows computing the frequency contents of *x(n)*, sampled at a frequency rate of *f_s_*, at a dense set of *L* frequencies in the range covered by the arc of the unit circle that begins at *ω_0_ = 2π f_0_* and ends at *ω_1_ = 2π f_1_* [17]:

(1) $$X(k)=\sum_{n=0}^{N-1}x(n)Z_{L}^{\mathrm{kn}}$$

In Equation (1) the transformation kernel $Z_{L}^{\mathrm{kn}}$ is given by (2).

(2) $$Z_{L}^{\mathrm{kn}}=\text{exp}\left\{-j\frac{2\pi \;n}{f_{s}}\left[f_{0}+\frac{\left(f_{1}-f_{0}\right)k}{L}\right]\right\}=\text{cos}(\omega n)-\mathrm{jsen}(\omega n)$$

$$\mathrm{where}\;\omega =\frac{2\pi }{f_{s}}\left[f_{0}+\frac{\left(f_{1}-f_{0}\right)k}{L}\right]\;\mathrm{and}\;k=0,1,\ldots ,L-1$$

The transformation kernel $Z_{L}^{\mathrm{kn}}$ can be implemented as two discrete recursive functions described by Equations (3) and (4) for its real *Z_R_* and imaginary *Z_I_* components, respectively:

(3) $$Z_{R}\;(n)=Z_{R}\;(n-1)\;\text{cos}(\omega n)-Z_{I}\;(n-1)\;\text{sin}(\omega n)$$

(4) $$Z_{I}\;(n)=Z_{I}\;(n-1)\;\text{cos}(\omega n)+Z_{R}\;(n-1)\;\text{sin}(\omega n)$$

### 2.2. Power Spectrum Analysis

The power spectrum of the CZT *X(k)* is given by (5):

(5) $${\left[X\left(k\right)\right]}^{2}={\left[X_{R}\left(k\right)\right]}^{2}+{\left[X_{I}\left(k\right)\right]}^{2}$$

From Equation (5), the minimum value of [*X(k)*]^2^ at which the CZT converges, on the high-resolution power spectrum, corresponds to the main frequency component of the discrete signal *x(n)*. The frequency resolution *Δ f* of the CZT power spectrum depends on the length of the analyzed unit circle arc, and the number of frequency elements covered in this length *L*, as given in Equation (6):

(6) $$\Delta \;f=\frac{\left(f_{1}-f_{0}\right)}{L}$$

### 2.3. CZT Computation Unit

**Figure 2.**
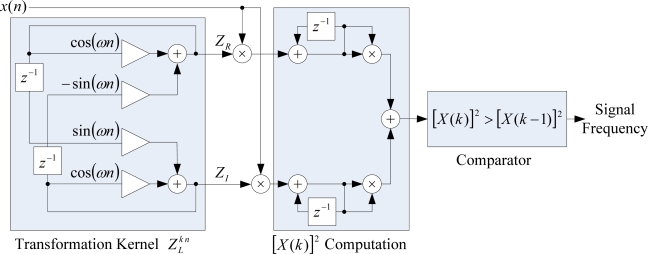
Operational structure for the CZT computation unit.

The operational architecture for the CZT computation unit of the proposed smart sensor is depicted in Figure 2. The transformation kernel block provides the real and imaginary components *Z_R_* and *Z_I_* of $Z_{L}^{\mathrm{kn}}$ implementing the recursive functions in Equations (3) and (4), respectively. The power spectrum magnitude [*X(k)*]^2^ is obtained by adding the squared accumulation of *Z_R_* and *Z_I_* times the discrete input signal *x(n)*. Finally, a magnitude comparison is carried out in order to identify the signal frequency with high resolution. The structure in Figure 2 for the CZT computation utilizes basic operations like addition, multiplications, and accumulations that provide an efficient architecture for hardware implementation.

### 2.4 Computational Complexity Comparison between CZT, FFT, and Zoom-FFT

In order to find an optimal algorithm that meets the international standard for frequency measurement [2], a computational complexity comparison between CZT, FFT, and Zoom-FFT, based on the number of operations, is developed in this section. The number of operations *Op_FFT_* for computing the FFT is given by Equation (7) [17]; whereas for computing the Zoom-FFT the number of operations *Op_ZFFT_* is given by Equation (8) [18]; finally the CZT requires *Op_CZT_* operations, as given by Equation (9) [17]. *N* is the data length for all algorithms, and *L* is the length of the interest region for the Zoom-FFT and the CZT:

(7) $${\mathrm{Op}}_{\mathrm{FFT}}=N{\text{log}}_{2}\;N$$

(8) $${\mathrm{Op}}_{\mathrm{ZFFT}}=N\left(1+{\text{log}}_{2}\;L\right)$$

(9) $${\mathrm{Op}}_{\mathrm{CZT}}=N\cdot L$$

Table 1 summarizes the computational complexity and acquisition time *T_acq_* required for complying international standard of frequency measurement with a resolution of *Δ f* = 0.01 Hz. The resolution *Δ f* for the FFT and the Zoom-FFT is given by Equation (10) with a sampling rate set to *f_s_* = 655.36 Hz; whereas the resolution for the CZT is given by Equations (2) and (6) with a sampling rate set to *f_s_* = 512 Hz in order to meet the standard.

(10) $$\Delta \;f=\frac{f_{s}}{N}$$

From Table 1 it can be seen that the CZT requires less operations for computation than FFT and Zoom-FFT, but the most restricting parameter is the acquisition time that according to the standard is 10 s. which is only met by CZT.

## 3. Simulation Results

The performance of the proposed smart sensor for real-time high-resolution frequency measurement is tested in this section. The simulation accuracy tests consist in feeding an artificially generated waveform into the proposed smart sensor for confirming its performance and compliance of the international standard CEI/IEC 61000-4-30 [2]. The tests carried out detect the frequency in high resolution of: a pure periodic signal, a periodic signal plus white noise, a main periodic signal with harmonic contamination, and a main periodic signal with harmonic contamination plus white noise.

### 3.1. Pure periodic signal

**Figure 3.**
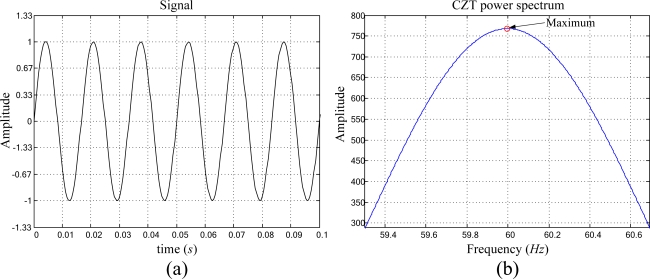
(a) Artificially generated pure periodic signal, (b) CZT power spectrum.

The analyzed pure periodic signal is described in Equation (11), with normalized amplitude, and frequency *f* = 60 Hz, as shown in Figure 3a. Analyzing the artificially generated pure periodic signal and utilizing the proposed smart sensor for high-resolution frequency estimation, the CZT power spectrum in Figure 3b is obtained, giving the frequency estimation result of 60.0000 Hz for *x(t)*:

(11) x(t)=sin(2πft)

### 3.2. Periodic Signal with White-Noise Contamination

**Figure 4.**
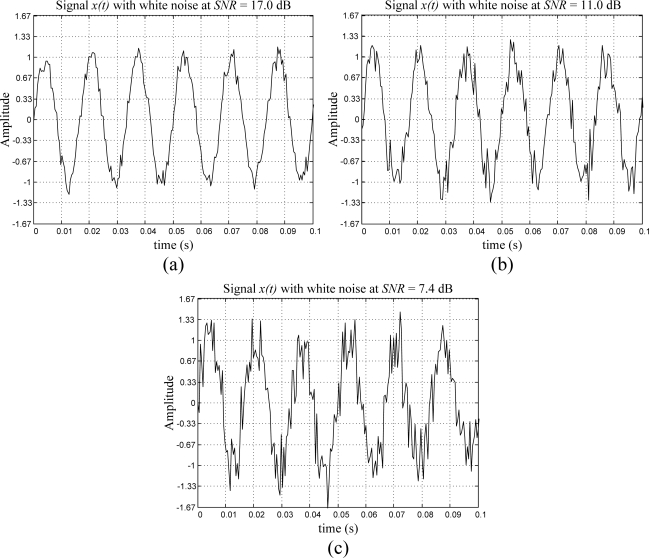
Artificially generated periodic signal with added white noise at *SNR* = (a) 17.0 dB, (b) 11.0 dB, (c) 7.4 dB.

The periodic signal contaminated with white noise is described in Equation (12). The amplitude of the periodic signal is normalized, and it has a frequency *f* = 60 Hz, whereas three cases of severe white noise *n(t)* contamination are considered. The first case considers a normalized periodic signal with white noise contamination at 10% of its amplitude for a *SNR =* 17.0 dB, as shown in Figure 4a. The second case, shown in Figure 4b, analyzes a normalized periodic signal with white noise contamination at 20% of the signal amplitude for a *SNR =* 11.0 dB. Finally, the third case considers a white noise contamination at 30% of the periodic signal amplitude for a *SNR =* 7.4 dB, presented in Figure 4c. Table 2 shows the obtained result from the proposed smart-sensor frequency estimation for 40 runs of the periodic signal with added white noise.

(12) x(t)=sin(2π f t)+n(t)

### 3.3. Periodic Signal plus Harmonic Contamination

**Figure 5.**
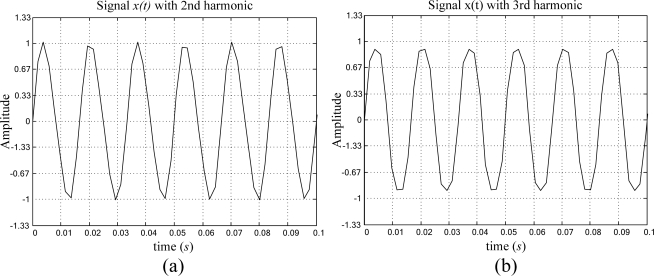

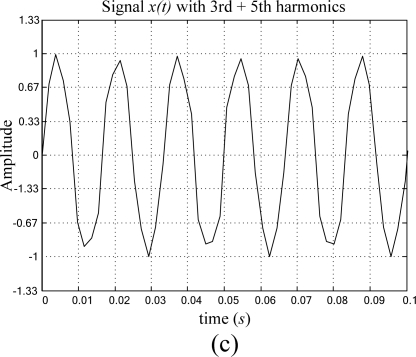
Artificially generated periodic signal contaminated with (a) 2^nd^, (b) 3^rd^, (c) (3^rd^ + 5^th^) harmonics, at 10% of the main periodic signal amplitude for a *SHR* = 20.0 dB.

The harmonic contamination of the periodic signal is described in Equation (13). Three different cases of harmonic contamination are treated. The first case considers a periodic signal contaminated with its 2^nd^ harmonic, as depicted in Figure 5a. The second case analyzes the periodic signal plus its 3^rd^ harmonic, as shown in Figure 5b. The last case considers the contamination of the periodic signal with its 3^rd^ + 5^th^ harmonics, as presented in Figure 5c. The amplitude of the periodic signal is normalized and has a frequency *f = 60* Hz, whereas the harmonic signals *h(t)* added to the periodic signal have an amplitude of 10% of the main periodic signal amplitude for a signal-to-harmonic ratio *SHR* = 20.0 dB. Table 3 shows the obtained result from the proposed smart-sensor frequency estimation for 40 runs of a periodic signal with harmonic contamination.

(13) $$\begin{array}{l} x(t)=\text{sin}\left(2\pi \;f\;t\right)+h(t)\;\mathrm{where}\\ h(t)=0.1\text{sin}(2\pi \;f\;l\;t)\;\mathrm{and}\;l=2,\;3,\;5 \end{array}$$

### 3.4. Periodic Signal plus White Noise and Harmonic Contamination

**Figure 6.**
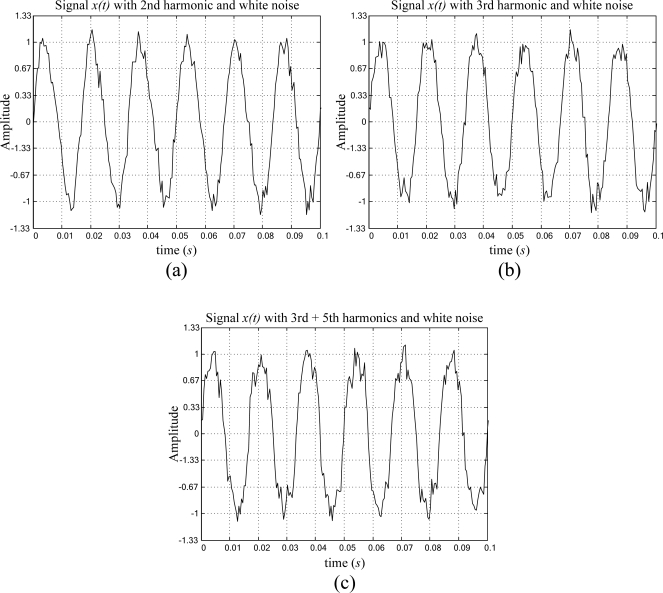
Artificially generated periodic signal contaminated with white noise, and (a) 2^nd^, (b) 3^rd^, (c) (3^rd^ + 5^th^) harmonics for a *SHNR* = 15.2 dB.

The white noise *n(t)* and harmonic *h(t)* contamination of a periodic signal is described in (14), where the amplitude of the periodic signal is normalized, and has a frequency *f = 60* Hz, whereas the white noise signal *n(t)* added to the periodic signal have a maximum amplitude of 10% of the main periodic signal amplitude and three different cases of harmonic contamination are considered. The first case considers a periodic signal contaminated with its 2^nd^ harmonic, as depicted in Figure 6a. The second case analyzes the periodic signal plus its 3^rd^ harmonic, as shown in Figure 6b. The last case considers the contamination of the periodic signal with its 3^rd^ + 5^th^ harmonics, as presented in Figure 6c. Where the signal-to-harmonic-plus-noise ratio *SHNR* = 15.2 dB. Table 4 shows the obtained result from the proposed smart-sensor frequency estimation for 40 runs of a periodic signal with white noise plus harmonic contamination.

(14) $$\begin{array}{l} x(t)=\text{sin}\left(2\pi \;f\;t\right)+h(t)+n(t)\;\mathrm{where}\\ h(t)=0.1\text{sin}(2\pi \;f\;l\;t)\;\mathrm{and}\;l=2,3,5 \end{array}$$

### 3.5. Simulation of an Instantaneous Jump on the Power Line Frequency

**Figure 7.**
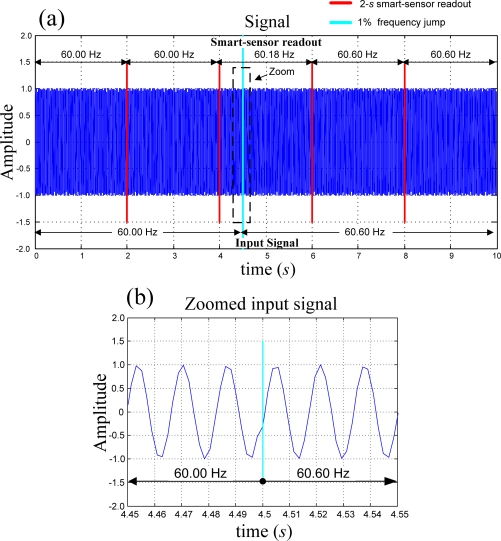
Instantaneous 1% frequency jump on the power line (a) comparison of the input-signal frequency against the smart-sensor readout, (b) zoomed input signal.

Figure 7 shows the effects of an instantaneous jump in the power line frequency over the proposed smart sensor readouts. The input signal was originally synthesized as a pure sinusoidal with a frequency of 60.0 Hz and at half of the measurement process the frequency was suddenly changed to 60.6 Hz, representing a 1% instantaneous jump. The international standard for frequency measurement [2], requires to give a readout every 10 *s*; the processing of the signal takes 1.0078 s and the instrument was set to provide a readout every 2 *s*. Figure 7a compares the input signal frequency against the smart-sensor readouts before and after the 1% frequency jump during the 10 s of the measurement required by the standard. Before the jump the frequency readout of the smart-sensor is 60.0 Hz as expected; during the jump, the readout is 60.18 Hz; once the signal frequency stabilizes, the readout is 60.6 Hz that corresponds to the magnitude of the jump. The final readout of the smart sensor follows the frequency change in the standard required time.

## 4. Experimental Results

This section presents the application of the proposed smart sensor for frequency estimation in two different study cases. The first study case considers the frequency measurement of the electrical power supply, and the second one considers the frequency measurement of the signal obtained from a Stanford Research DS345 synthesized function generator [19]. Some of the main features of this device are that it generates standard waveforms with frequency resolution of 1 *μ*Hz, and accuracy of ±5 part-per-million; which makes it suitable for being used as reference to test the performance of the proposed smart sensor. The output from the synthesized function generator is contaminated with white noise at 10%, 20%, and 30% for a *SNR* = 17.0, 11.0, 7.4 dB, respectively. The proposed smart sensor depicted in Figure 2 is implemented in a low cost FPGA device xc3s1000 from Xilinx, embedded on the Spartan-3 Starter Board from Digilent [20]. The processing time takes 1.0078 s to perform the frequency estimation and the smart sensor provides readouts every 2 *s*.

### 4.1. Power Supply Frequency Measurement

**Figure 8.**
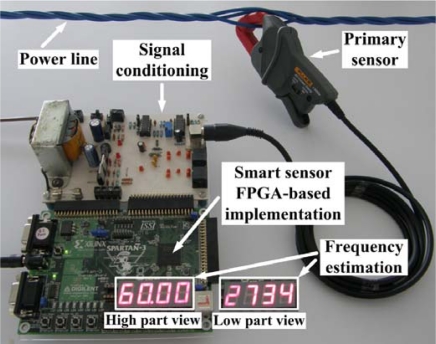
Experiment setup for high-resolution power line frequency estimation utilizing an FPGA-implementation of the proposed smart sensor.

**Figure 9.**
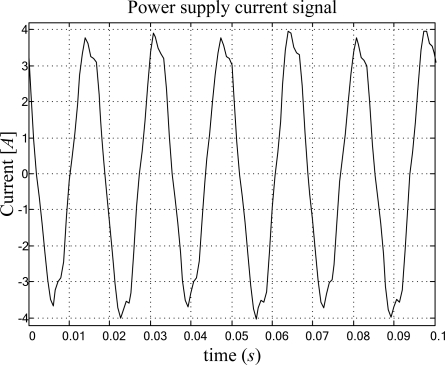
Power supply current.

The proposed smart sensor for frequency measurement can be utilized with several primary sensors. For this study case, the standard off-the-shelf current clamp i200s from Fluke is utilized for current monitoring. A 12-bit 4-channel serial-output analog to digital converter ADS7841 [21] is used for signal acquisition. Figure 8 shows the experiment setup for high-resolution frequency measurement monitoring the power-line-supply current from a standard electrical load (1 *hp* induction motor) with a current clamp as primary sensor. The current signal is sensed during 1 s at a sampling frequency of 512 Hz. The signal conditioning system provides the FPGA implementation of the proposed smart sensor with 512 discrete samples for high-resolution frequency estimation, the result is shown on the 4-digit 7-segment LED display, where the user selects between the high- and low-part of the estimation result to be shown on the display. For the case treated here 60.002734 Hz. Figure 9 shows the sensed current signal from the power line. By taking 40 readouts of the power-line frequency, the calculated mean and standard deviation are 60.0008 Hz and 0.0013 Hz, respectively.

### 4.2. Frequency Estimation of Digitally Synthesized Functions

**Figure 10.**
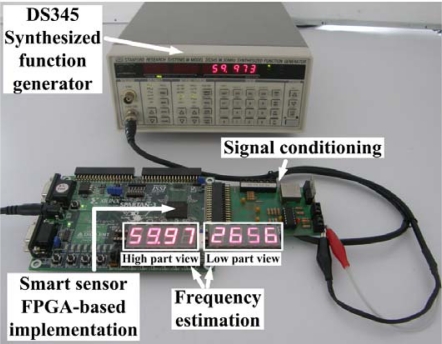
Experiment setup for high-resolution frequency estimation monitoring voltage signals and utilizing the FPGA-implementation of the proposed smart sensor.

**Figure 11.**
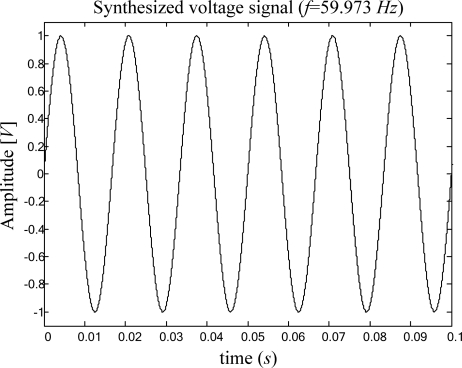
Pure sinusoidal signal with frequency of 59.973 Hz generated by the Stanford Research DS345 synthesized function generator.

In this case the frequency is estimated from voltage signals generated by a Stanford Research DS345 synthesized function generator. Similar to the previous case of study, a 12-bit 4-channel serial-output analog to digital converter ADS7841 is used for signal acquisition. Figure 10 shows the experiment setup for high-resolution frequency measurement, monitoring voltage signals. The synthesized voltage signal shown in Figure 11 is sampled during 1 s at a sampling rate of 512 Hz. The signal conditioning system provides the FPGA implementation of the proposed smart sensor with 512 discrete samples for high-resolution frequency estimation. The estimation result is shown on the 4-digit 7-segment LED display, where the high- and low-part of the estimation result can be selected for being shown on the display. For the case treated here, the frequency estimation of a synthesized pure sinusoidal signal with a frequency of 59.973 Hz is 59.972656 Hz.

**Figure 12.**
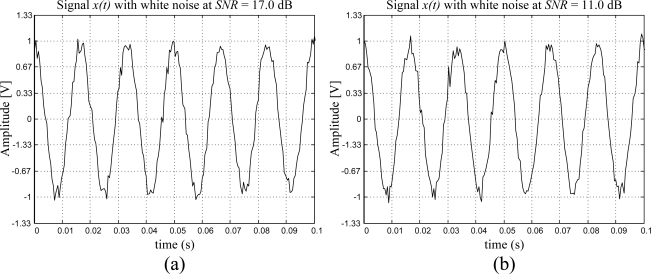

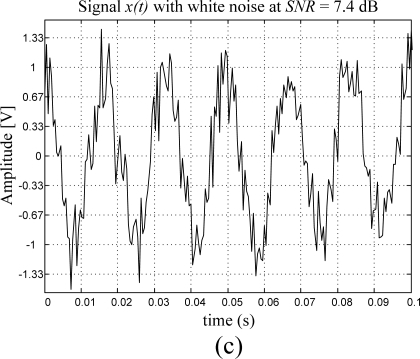
60 Hz Sinusoidal signals with white noise contamination at *SNR* = (a) 17.0 dB, (b) 11.0 dB, (c) 7.4 dB, generated by the Stanford Research DS345 synthesized function generator.

To test the performance of the proposed smart sensor, three experiments are set up considering a 60 Hz sinusoidal signal generated by the DS345 function generator with white noise contamination at 10%, 20%, and 30% of its amplitude for a *SNR* = 17.0, 11.0, 7.4 dB, and as shown in Figure 12a–c, respectively. Table 5 summarizes the obtained results for 40 readouts from the proposed smart sensor.

### 4.3. Smart Sensor Linearity

**Figure 13.**
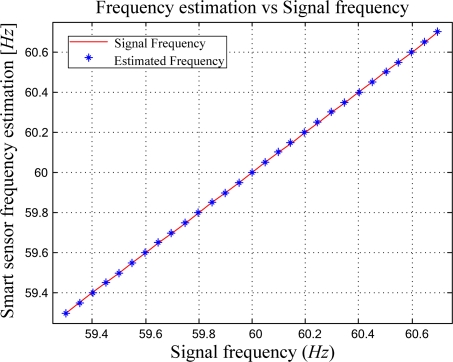
Linearity test of the proposed smart sensor for high-resolution frequency estimation.

A test of linearity to the proposed smart sensor for high resolution frequency estimation is carried out in this section. Figure 13 shows the proposed smart sensor behavior under small variations of the input signal frequency. The input signal frequency increases from 59.3 Hz in steps of 0.05 Hz up to 60.7 Hz obtaining the correspondence line in Figure 13; which shows a linear behavior of the proposed smart sensor. The frequency variation range from 59.3 Hz to 60.7 Hz corresponds to the international standard CEI/IEC 61000-4-30 [2]; which states that the power frequency of the supply signal must vary around ±1% (from 59.4 Hz to 60.6 Hz). The results show that the proposed smart sensor provides a very accurate solution with an uncertainty of ±0.0024 Hz, with a maximum estimation error of 0.0055 Hz and an integral linearity of 0.189 %.

## 5. Discussion

An important result of this work is the hardware implementation of a smart sensor for high-resolution frequency detection in real-time applications. The proposed smart sensor is implemented in a low-cost FPGA device reaching operation speeds of 33.74 *M*Hz; which provides accurate frequency readouts every 2 *s*.

The proposed smart sensor shows high performance operation, meeting the international standards for frequency measurement even with high-level noise contamination. This is shown in the simulation results where different study cases were treated, with the white-noise-contamination case at *SNR* = 7.4 dB being the one with the highest error. On the other hand, the proposed sensor shows a very good performance in analyzing real signals generated with a highly accurate reference source with high resolution, giving mean errors of 0.0007, 0.0001, and 0.0020 Hz for a periodic signal contaminated with white noise at *SNR* = 17.0, 11.0, and 7.4 dB, respectively. Regarding harmonic contamination, the proposed sensor showed to be highly insensitive as depicted in Tables 3 and 4. Finally, the proposed smart sensor shows a linear behavior for small frequency variations, which means that it will follow any small change in the signal frequency; this is shown in the linearity tests where the efficiency of the proposed smart sensor for power systems frequency estimation is demonstrated, providing results that are even below of those prescribed by international standards.

## 6. Conclusions

This work presents the development of a smart sensor for frequency estimation in real-time applications. The proposed sensor complies with international standards for frequency measurement in power systems, providing fast and accurate estimations with high resolution and small deviations as demonstrated by the results obtained in linearity test section, different from other methodologies where low resolutions of just decades of Hz are achieved. The proposed smart sensor implementation uses a simple architecture describing recursive functions for the CZT computation utilizing addition, multiplication, and accumulation operations, different from other algorithms using complex mathematics (e.g., FFT) and weighting factors for increasing their resolution (e.g., Zoom-FFT) or implementing the CZT in multiple iterations. The proposed system is considered a smart sensor since it integrates a commercially available current clamp, hall-effect sensor, or resistor as primary sensor, analyzing the corresponding output with digital signal processing techniques in the time domain for estimating the input signal frequency online, with high resolution. Other techniques can use only one kind of primary sensor and require the transformation of the monitored signal into the frequency domain. From the stated above, it can be concluded that the proposed smart sensor for high resolution frequency estimation is a low-cost and efficient solution for real-time application in power systems such as control and protection, thanks to its straightforward FPGA-based implementation that provides an accurate frequency readout every 2 s. complying international standards for power frequency monitoring different from other that either have problems meeting the norm [9–11], or require complex algorithms that make difficult their implementation [22,23].

## Figures and Tables

**Table 1.** Estimated number of operations and acquisition time comparison.

**Parameter**	**FFT**	**Zoom-FFT**	**CZT**

*N*	65,536	65,536	512
*L*	--	512	512
*Op*	1,048,576	655,360	262,144
*T_acq_* (*s*)	100	100	1

**Table 2.** Proposed smart-sensor frequency estimation of a periodic signal with added white noise at *SNR* = 71.0 dB, 11.0 dB, and 7.4 dB.

**Signal + white noise at *SNR* (dB)**	**Frequency estimation (Hz)**		**Error (Hz)**	

	**Mean (μ)**	**Standard deviation (σ)**	**Mean (μ)**	**Standard deviation (σ)**

17.0	59.9997	0.0040	0.0003	0.0040
11.0	59.9992	0.0066	0.0008	0.0066
7.4	60.0007	0.0106	0.0007	0.0106

**Table 3.** Proposed smart-sensor frequency estimation of a periodic signal contaminated with its 2^nd^, 3^rd^, and (3^rd^ + 5^th^) harmonics, at 10% of the main periodic signal amplitude for a *SHR* = 20.0 dB.

**Signal + harmonic**	**Frequency estimation (Hz)**		**Error (Hz)**	

	**Mean (μ)**	**Standard deviation (σ)**	**Mean (μ)**	**Standard deviation (σ)**

2^nd^	60.0000	0.0000	0.0000	0.0000
3^rd^	60.0000	0.0000	0.0000	0.0000
3^rd^ + 5^th^	60.0000	0.0000	0.0000	0.0000

**Table 4.** Proposed smart-sensor frequency estimation of a periodic signal contaminated with white noise and its 2^nd^, 3^rd^, and (3^rd^ + 5^th^) harmonics for a *SHNR* = 15.2 dB.

**Signal + white noise and harmonic**	**Frequency estimation (Hz)**		**Error (Hz)**	

	**Mean (μ)**	**Standard deviation (σ)**	**Mean (μ)**	**Standard deviation (σ)**

2^nd^	60.0002	0.0042	0.0002	0.0042
3^rd^	59.9996	0.0032	0.0004	0.0032
3^rd^ + 5^th^	59.9999	0.0038	0.0001	0.0038

**Table 5.** Frequency estimation of a 60 Hz sinusoidal signal contaminated with white noise at *SNR* = 17.0 dB, 11.0 dB, and 7.4 dB.

**Signal + white noise at *SNR* (dB)**	**Frequency estimation (Hz)**		**Error (Hz)**	

	**Mean (μ)**	**Standard deviation (σ)**	**Mean (μ)**	**Standard deviation (σ)**

17.0	60.0007	0.0034	0.0007	0.0034
11.0	60.0001	0.0064	0.0001	0.0064
7.4	60.0020	0.0091	0.0020	0.0091
